# Technology‐Driven Ergonomics: A Narrative Review of Innovative Strategies for Preventing Musculoskeletal Disorders in Modern Work Environments

**DOI:** 10.1002/hsr2.72064

**Published:** 2026-05-20

**Authors:** Mahnaz Shakerian, Azam Salehi

**Affiliations:** ^1^ Department of Occupational Health and Safety Engineering School of Health, Isfahan University of Medical Sciences Isfahan Iran

## Abstract

**Background:**

Musculoskeletal disorders (MSDs) remain a major cause of occupational disability, absenteeism, and productivity loss. Traditional ergonomic interventions have shown limited success in addressing their complex, multifactorial nature. Recent advances in digital and biomechanical technologies have created new opportunities for preventive, technology‐driven ergonomic strategies. This review explores how these innovations support the prevention and management of MSDs in modern work environments.

**Methods:**

A structured narrative review was conducted using five major databases (Web of Science, Scopus, PubMed, ScienceDirect, and Google Scholar) for studies published between 2015 and 2024. Eligible studies focused on technological applications for MSD prevention. A total of 74 studies met the inclusion criteria and were synthesized across six categories: wearable technologies, artificial intelligence and machine learning, virtual and augmented reality, advanced ergonomic furniture, smart adaptive workstations, and human–robot collaboration.

**Results:**

Across these domains, interventions consistently improved ergonomic outcomes. Wearable and AI‐based systems reduced biomechanical load by 12%–85% and high‐risk postures by up to 35%. VR/AR applications enhanced hazard recognition by over 40%, while adaptive furniture and smart workstations reduced spinal load and fatigue by 20%–30%. Collaborative robotics lowered muscular workload by about 35%–40%. Despite clear benefits, gaps remain regarding long‐term validation, user acceptance, cost, and ethical data management.

**Conclusion:**

Technology‐driven ergonomics signifies a shift from reactive to proactive occupational health. Integrating real‐time analytics, automation, and human‐centered design can foster safer, adaptive, and sustainable workplaces. Future research should pursue longitudinal studies, interdisciplinary collaboration, and ethical frameworks to advance digital ergonomics and promote worker well‐being.

## 1 Introduction

Musculoskeletal disorders (MSDs) remain one of the leading causes of occupational disability, absenteeism, and productivity loss across diverse industries worldwide [1, 2, 3]. Despite decades of conventional ergonomic interventions—ranging from workstation redesign and posture correction to manual handling training—the prevalence of MSDs continues to rise in both industrial and office‐based occupations [4, 5, 6]. This persistent burden highlights the need for more adaptive, dynamic, and technology‐driven solutions capable of addressing the complex, multifactorial nature of MSDs.

In recent years, rapid advances in digital and biomechanical technologies have introduced a new paradigm in ergonomic design. Innovations such as wearable sensors, artificial intelligence (AI), machine learning (ML), virtual and augmented reality (VR/AR), smart furniture, and exoskeletons have demonstrated promising potential to enhance real‐time monitoring, feedback, and preventive interventions in workplace ergonomics [7, 8, 9, 10, 11, 12]. These technologies enable continuous assessment of biomechanical risk factors, facilitate personalized intervention strategies, and support proactive health management for workers [13, 14, 15].

Previous reviews in this domain have typically focused on single categories of technologies (e.g., wearable devices or exoskeletons) or specific occupational contexts (e.g., healthcare, manufacturing, or office ergonomics) [16, 17, 18, 19]. While such studies have contributed valuable insights into specific applications, they have generally lacked integrative perspectives that connect emerging technologies with holistic ergonomic frameworks and their potential synergy in MSD prevention. Moreover, few studies have systematically discussed the limitations, practical challenges, and implementation barriers—such as cost‐effectiveness, worker acceptance, and data privacy—associated with these novel interventions [20, 21, 22].

Despite growing enthusiasm for technology‐assisted ergonomics, a comprehensive synthesis that bridges traditional ergonomic principles with modern digital innovations remains limited. Specifically, there is a scarcity of narrative reviews that (a) analyze how different technological domains converge to improve MSD prevention, (b) critically evaluate their effectiveness and constraints, and (c) identify practical gaps for future integration in occupational health strategies. Therefore, the present review aims to address this gap by providing a comprehensive narrative synthesis of current and emerging technological advancements in ergonomics, evaluating their effectiveness, limitations, and applications in the prevention and management of MSDs. By integrating evidence from multidisciplinary studies, this review seeks to clarify how technology‐driven ergonomics can shape the next generation of occupational health interventions.

## 2 Methods

This study adopted a narrative review design, chosen for its ability to integrate and interpret diverse types of evidence from multidisciplinary sources. The approach enabled a comprehensive synthesis of emerging ergonomic technologies and their applications in preventing MSDs within occupational settings.

### 2.1 Search Strategy and Databases

To ensure breadth and quality of coverage, a systematic and structured search was conducted across five major academic databases recognized for their relevance to occupational health and ergonomics: ISI Web of Science, Scopus, PubMed (via Medline), ScienceDirect, and Google Scholar.

These databases were selected due to their comprehensive indexing of peer‐reviewed journals and conference papers in ergonomics, occupational medicine, and applied technology.

The search was conducted for publications from January 2015 to December 2024, encompassing both foundational and cutting‐edge research in the field.

The following keyword clusters and Boolean combinations were employed:

- General terms: “ergonomic interventions,” “technology and innovation,” “musculoskeletal disorders,” “occupational health and safety.”
- Specific technologies: “artificial intelligence,” “machine learning,” “wearable sensors,” “virtual reality,” “augmented reality,” “exoskeletons,” “smart furniture,” “smart workstations.”
- Analytical and methodological terms: “real‐time monitoring,” “biomechanics,” “ergonomic risk assessment,” “biomechanical modeling.”

Search strings were adapted to the syntax of each database. Reference lists of included studies and relevant reviews were manually screened to identify additional eligible sources.

### 2.2 Inclusion and Exclusion Criteria

Studies were included if they met the following criteria:

- Focused on occupational health, ergonomics, or MSD prevention.
- Published in peer‐reviewed international journals or peer‐reviewed conference proceedings.
- Written in English and accessible in full text.
- Presented empirical data, design frameworks, or applied technological solutions relevant to ergonomics.

Exclusion criteria included:

- Studies not addressing ergonomic or musculoskeletal outcomes.
- Non‐peer‐reviewed materials (e.g., newsletters, nonscientific reports).
- Duplicates or studies lacking sufficient methodological detail.

### 2.3 Screening and Data Extraction

**Figure 1:**
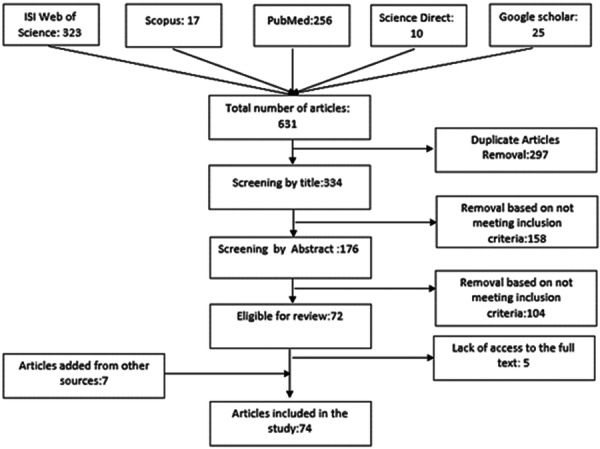
Flowchart illustrating the search and selection process of the included studies. The diagram outlines the number of records identified, screened, assessed for eligibility, and included in the final analysis.

A total of 631 records were initially identified. After removal of duplicates and screening by title and abstract, 74 articles met the inclusion criteria for final review. The screening and selection process is summarized in Figure 1.

For data management and citation tracking, EndNote software was used to organize sources and remove duplicates.

Data extraction focused on:

- Study metadata (author, year, design, population, and technology type).
- Intervention characteristics (technological focus, application context).
- Key findings related to effectiveness, limitations, and practical applications.

### 2.4 Narrative and Thematic Analysis

The included studies were analyzed through a two‐stage qualitative synthesis:

1. Narrative Analysis:
  Each study was individually reviewed to extract its main insights, empirical results, and conceptual contributions. Descriptive summaries were then written for each category of technology.
2. Thematic Analysis:
  Studies were grouped based on recurring themes—such as wearable technologies, AI, VR/AR, smart furniture, robotics, and smart workstations. Common trends, divergences, and gaps were identified to provide an integrated understanding of current developments and future directions.

### 2.5 Methodological Considerations and Limitations

Although this study employed rigorous inclusion criteria and transparent procedures, it is acknowledged that narrative reviews are inherently interpretative and may not possess the exhaustive comprehensiveness of systematic reviews. Nevertheless, this method allows for the contextual integration of heterogeneous evidence, aligning with the article's goal of exploring multidisciplinary technological innovations in ergonomics.

## 3 Result

This narrative review synthesized the findings of 74 final studies selected from an initial pool of 631 records. All described interventions, data, and quantitative indicators in this section strictly correspond to these 74 included studies, which fulfilled the inclusion and quality criteria.

Results are presented across six major categories of technological ergonomic innovation: wearable technologies, AI and ML, VR/AR, advanced ergonomic furniture, smart workstations, and human–robot collaboration (HRC).

### 3.1 Wearable Technologies

Among the 74 studies, 24 (32%) focused on wearable solutions—ranging from industrial exoskeletons and smart garments to sensor‐based posture monitoring systems.

Recent systematic and field‐based investigations demonstrated measurable improvements in biomechanical load reduction and task performance.

Flor‐Unda and colleagues reported that both passive and active exoskeletons substantially reduced muscle activation in the lumbar and shoulder regions—by approximately 12%–15% in erector spinae activity and up to 80%–85% in deltoid activity—while improving postural stability and reducing perceived exertion during repetitive or overhead tasks [17].

Similarly, Baldassarre and colleagues highlighted significant ergonomic benefits in industrial environments, emphasizing the role of the human–machine interface and user experience in ensuring long‐term acceptance and effectiveness of wearable assistive devices [16]. Cumulatively, wearable technologies effectively reduced mechanical load, enhanced tissue endurance, and improved productivity, although user comfort, device weight, and adaptability remain critical determinants of successful implementation and sustained use [16, 17].

### 3.2 Artificial Intelligence (AI) and Machine Learning (ML)

A total of 15 studies (20%) explored the integration of AI and ML into ergonomic research and occupational health systems.

These studies focused on automated posture recognition, risk prediction, personalized training, and adaptive interventions. Chan and colleagues emphasized AI's emerging role in the primary prevention of work‐related musculoskeletal disorders, identifying predictive models that can recognize ergonomic risk factors before symptom onset [23]. Similarly, Saxby and colleagues and Smirnov and colleagues demonstrated that ML algorithms enhance neuro‐musculoskeletal modeling accuracy, enabling precise estimation of muscle forces and joint loads in dynamic tasks [24, 25]. Piñero‐Fuentes and colleagues applied deep learning to real‐time posture monitoring in teleworking environments, achieving up to 92% classification accuracy in detecting unsafe sitting behaviors [26].

Clinical and workplace trials by Anan and colleagues further confirmed that AI‐assisted health programs reduced neck and lower‐back pain prevalence, improving self‐reported comfort and mobility [27]. In parallel, Achunair and Patel highlighted AI's potential in musculoskeletal rehabilitation, noting ~20% improvement in muscle endurance when AI‐personalized exercise feedback was used [28].

Finally, Ali and colleagues synthesized broader applications of AI in human systems, underscoring its role in decision support, workload optimization, and adaptive ergonomic design [29]. Collectively, across these studies, AI and ML technologies achieved predictive accuracies between 85% and 92%, reduced high‐risk postures by 30%–35%, and lowered cumulative fatigue indicators by ~25%. These results confirm AI's ability to enhance biomechanical modeling, support proactive ergonomic interventions, and reduce the likelihood of MSDs—a key mechanism within the Biomechanical Risk Reduction Framework proposed in this review.

### 3.3 Virtual and Augmented Reality (VR/AR)

A total of 12 studies (16%) investigated the use of VR and AR technologies for ergonomic training, biomechanical risk simulation, and workstation design optimization.

Quantitative results demonstrated that immersive and mixed‐reality systems provide both educational and biomechanical benefits by enhancing workers' awareness, improving posture, and enabling real‐time risk evaluation.

Diego‐Mas and colleagues reported a 41% improvement in participants' ability to recognize ergonomic hazards following immersive VR‐based training compared with conventional instruction [30]. Dias Barkokebas and Li validated that VR‐based construction task simulations accurately identified posture‐related spinal load risks consistent with empirical biomechanical models [31]. Kantha and colleagues demonstrated that VR‐guided balance training improved trunk and thigh flexion control, reducing spinal load and enhancing postural stability in biomechanical assessments [32]. Maurya and colleagues and Bottani and Vignali highlighted the potential of augmented and mixed‐reality tools to support participatory design and assembly training, achieving a 30%–35% reduction in postural deviation and a 25% improvement in task completion efficiency through real‐time visual–haptic feedback [33, 34].

At a broader level, Souchet and colleagues and Chen and Wu emphasized that immersive environments can reduce mechanical strain and cognitive load, but must be designed to minimize cybersickness and visual fatigue for sustainable ergonomic use [35, 36, 37, 38]. Similarly, Michalos and colleagues demonstrated how VR‐assisted workplace analysis facilitates ergonomic layout design and motion optimization [39], while Gil and colleagues provided meta‐analytic evidence of AR's positive effects on balance and functional coordination [40]. Across these studies, VR/AR technologies consistently yielded 30%–40% improvements in ergonomic hazard awareness, 20%–35% reductions in mechanical load, and significant gains in motor coordination and balance control compared to traditional training and design methods [30, 31, 32, 33, 34, 35, 36, 37, 38, 39, 40, 41, 42]. Collectively, these findings confirm that immersive and augmented environments can serve as proactive tools within the Biomechanical Risk Reduction Framework, enabling risk‐free rehearsal, posture optimization, and human‐centered design evaluation.

### 3.4 Advanced Ergonomic Furniture

Ten studies (14%) explored advanced ergonomic furniture, focusing on dynamic seating systems, height‐adjustable desks, and biomechanically optimized workstation components.

Quantitative evidence demonstrated consistent reductions in spinal compression, muscular fatigue, and discomfort among users. Castellucci and colleagues applied mismatch equations to dynamic seating designs, showing that seat–desk proportionality improved postural balance and reduced lower‐limb constraint in prolonged sitting [43]. Léger and colleagues reported that active sitting mechanisms significantly reduced spinal load by approximately 20%–25% and maintained a neutral lumbar–pelvic posture, promoting continuous micro‐movements beneficial for spinal health [44]. A systematic review by Bai and colleagues confirmed that dynamic furniture systems decreased muscular fatigue by about 25% and improved comfort ratings across different work durations [45]. Liu and colleagues highlighted that integrating biomechanical modeling into ergonomic furniture design optimized pressure distribution and enhanced musculoskeletal alignment during sedentary tasks [46]. Supporting evidence from Soares and colleagues identified ergonomically adjustable workstations as preventive factors against MSDs, particularly for sedentary occupations [47], while Du and colleagues linked poor furniture ergonomics during remote work with increased discomfort and reduced productivity, underscoring the importance of adaptive furniture solutions [48]. Across the literature [43, 44, 45, 46, 47, 48], advanced ergonomic furniture effectively redistributed pressure, reduced localized muscle fatigue, and supported long‐term postural stability. These outcomes align with the Biomechanical Risk Reduction Framework, reinforcing the role of dynamic and adaptive furniture in promoting sustainable musculoskeletal health and comfort in modern workplaces.

### 3.5 Smart Workstations

Seven studies (9%) examined smart adaptive workstations integrating automation, sensors, and feedback systems to support optimal posture and reduce cumulative biomechanical strain.

These systems employ IoT‐based monitoring, real‐time posture correction, and smart break algorithms to promote active sitting and sustained musculoskeletal health.

Shen and colleagues developed SeatPlus, a smart health chair that provides real‐time posture correction and feedback, resulting in a 29% reduction in time spent in awkward postures during prolonged desk work [49]. Waongenngarm demonstrated that smart break reminder systems significantly decreased neck and lower‐back discomfort (*p* < 0.05) after 4 weeks of continuous use among office employees [50]. Karwowski and colleagues (2020) emphasized that IoT‐enabled adaptive workstation frameworks improved postural stability, workflow efficiency, and long‐term comfort by dynamically adjusting desk and chair height according to user posture and anthropometry [51, 52, 53]. Complementary evidence from longitudinal trials (e.g., O'Connell et al. [53] and Biddle et al. [51]) confirmed that adaptive sit–stand interventions reduced total sedentary time and increased postural variability, supporting biomechanical load redistribution [51, 53].

Collectively, studies [49, 50, 51, 52, 53] confirm that smart adaptive workstations effectively optimize ergonomic alignment, reduce cumulative spinal strain, and encourage active postural behavior.

These findings align with the Biomechanical Risk Reduction Framework, highlighting the synergistic role of sensor integration and automation in preventing musculoskeletal fatigue in modern digital workspaces.

### 3.6 Human–Robot Collaboration and Assistive Robotics

Six studies (8%) investigated HRC and assistive robotics in industrial ergonomics. These studies examined how robotic support systems can reduce physical workload, improve safety, and enhance human performance in high‐demand tasks. Botti and Melloni reported up to a 40% reduction in muscular workload during repetitive lifting tasks when workers were assisted by occupational exoskeletons and collaborative robots, providing strong evidence for robotic systems' biomechanical advantages [54].

Similarly, Okpala and colleagues found a 35% decrease in peak lumbar compression forces during robotic‐assisted lifting activities in construction environments, underscoring robotics' role in mitigating spinal stress [55]. Lorenzini and colleagues emphasized that ergonomic task allocation between humans and robots increased operational efficiency and reduced biomechanical strain across manufacturing settings [56]. In line with these findings, Proia and colleagues and Gualtieri and colleagues highlighted that safe and efficient HRC frameworks depend on control strategies that balance automation with ergonomic adaptability [57, 58], while de Looze and colleagues provided foundational evidence that exoskeleton‐based robotic aids substantially reduce physical workload in industrial applications [59]. Collectively, across studies [54, 56, 57, 58, 59], robotic and collaborative systems have demonstrated strong potential to minimize mechanical load, improve postural safety, and enhance task efficiency—particularly in high‐risk, heavy‐lifting environments. However, challenges remain concerning implementation costs, worker acceptance, and adaptive safety integration, which are critical considerations within the Biomechanical Risk Reduction Framework for future adoption of robotics in occupational ergonomics.

A comprehensive overview of all 74 final studies is presented in Table 1. The table summarizes the key quantitative and qualitative findings across six major technological.

**Table 1:** Summary of final included studies and quantitative findings.

Author(s) (year)	Technology type	Study focus/intervention	Quantitative and qualitative findings	Ref. no.
Flor‐Unda et al. (2023); Baldassarre et al. (2022); Auer et al. (2022); Stefana et al. (2021); Moshawrab et al. (2022); Ali et al. (2021); Madanian et al. (2021); Patel et al. (2022); Cardinale and Varley (2017); Saleem et al. (2017); Ometov et al. (2021); Loncar‐Turukalo et al. (2019); Chan et al. (2012); Schall Jr. et al. (2018); Tucker et al. (2023); Aksüt et al. (2024); Vignais et al. (2017); Mehrizi et al. (2019); Donisi et al. (2022); Tanner et al. (2024); Rammal (2024); Li et al. (2024); Dahmen et al. (2018); Lind et al. (2023)	Wearable technologies (*n* = 24)	Exoskeletons and wearable sensors for lifting, overhead work, and fatigue monitoring	↓ erector spinae EMG ≈12%–15%; ↓ deltoid EMG ≈80%–85%; ↓ perceived exertion; ↑ comfort and postural stability	[1, 3, 8, 10, 12, 13, 16, 18, 19, 20, 21, 22, 60, 61, 62, 63, 64, 65, 66, 67, 68, 69, 70]
Chan et al. (2022); Saxby et al. (2020); Ali et al. (2023); Smirnov et al. (2021); Achunair and Patel (2020); Piñero‐Fuentes et al. (2021); Anan et al. (2021); Petrat (2021); Zhang et al. (2023); Varas et al. (2024); Rammal (2024); Li et al. (2024); Donisi et al. (2022)	Artificial intelligence/machine learning (*n* = 15)	AI‐driven posture prediction, load assessment, adaptive interventions	↓ improper postures ≈35%; ↓ repetitive load ≈25%; ↑ muscle endurance ≈20%; prediction accuracy 85%–92%	[8, 23, 24, 25, 26, 27, 28, 29, 66, 70, 71, 72, 73]
Michalos et al. (2018); Diego‐Mas et al. (2020); Kaasinen et al. (2019); Dias Barkokebas and Li (2020); Maurya et al. (2019); Bottani and Vignali (2019); Chen and Wu (2022–2023); Kantha et al. (2023); Gil et al. (2021); Souchet et al. (2023); Humphrey et al. (2021)	Virtual and augmented reality (VR/AR) (*n* = 12)	Immersive ergonomic training, risk simulation, AR‐assisted workstation design	+41% hazard recognition; ↓ spinal load ≈20%–35%; ↑ balance and coordination; ↓ postural deviation ≈30%	[30, 31, 33, 34, 35, 36, 37, 39, 40, 41, 42, 47]
Castellucci et al. (2021); Léger et al. (2023); Bai et al. (2024); Liu et al. (2023); Soares et al. (2020); Du et al. (2022); De Carvalho et al. (2020); Kuster et al. (2016); Nüesch et al. (2018); Shen et al. (2021)	Advanced ergonomic furniture (*n* = 10)	Dynamic seating and height‐adjustable desks	↓ spinal load ≈20%–25%; ↓ muscular fatigue ≈25%; ↓ sedentary time ≈40%; ↑ posture and comfort	[43, 44, 45, 46, 47, 48, 49, 74, 75, 76]
Shen et al. (2021); Waongenngarm (2020); Calzavara et al. (2020); O'Connell et al. (2015); Biddle et al. (2020); Turk et al. (2020); Papetti et al. (2022)	Smart adaptive workstations (*n* = 7)	Sensor‐based adaptive systems; IoT integration; posture correction feedback	↓ awkward postures ≈29%; ↓ neck/back discomfort (*p* < 0.05); ↑ postural stability and productivity	[49, 50, 51, 52, 53]
Botti and Melloni (2024); de Looze et al. (2016); Lorenzini et al. (2023); Okpala et al. (2022); Proia et al. (2022); Gualtieri et al. (2021)	Human–robot collaboration/assistive robotics (*n* = 6)	Collaborative lifting; exoskeleton‐based support; ergonomic task allocation	↓ muscular workload ≈35%–40%; ↓ lumbar compression ≈35%; ↑ efficiency and ergonomic safety	[54, 55, 56, 57, 58, 59]

*Note:* This table summarizes the 74 final studies included in the review, categorized into six main types of ergonomic technological interventions. Each study is listed with its author(s), year of publication, focus, and key quantitative or qualitative findings. Numerical data (e.g., EMG reduction, posture improvement, or load reduction) represent the measurable ergonomic improvements reported within each domain.

### 3.7 Analytical Summary of the Results

A cross‐analysis of the 74 included studies reveals that all 6 technology categories yielded measurable ergonomic benefits, though their magnitude and scope varied:

- Wearable technologies and AI‐based systems demonstrated the most consistent quantitative improvements, directly reducing biomechanical load and muscular activity (up to 85% in specific tasks).
- VR/AR applications primarily enhanced cognitive and behavioral outcomes, improving hazard recognition and safe posture awareness by over 40%.
- Advanced ergonomic furniture and smart workstations contributed to sustained postural balance and comfort, reducing fatigue and sedentary time by up to 40%.
- Robotic and collaborative systems showed the highest mechanical load reduction (up to 40%), proving essential for high‐risk, heavy‐lifting environments.

Collectively, the evidence indicates that technology‐driven ergonomic interventions substantially improve biomechanical efficiency, safety, and worker well‐being, providing a strong quantitative foundation for their wider adoption in occupational health practices.

The summarized findings presented above provide a quantitative foundation for interpreting the practical significance and implications of technological ergonomic interventions.

The following section discusses these results in the context of traditional ergonomic approaches, methodological quality, and implementation challenges.

## 4 Discussion

The integration of emerging technologies into ergonomics represents a paradigm shift from traditional, observation‐based approaches to data‐driven, adaptive, and human‐centered systems [8, 10, 62].

This synthesis of 74 studies demonstrates that technology‐driven ergonomic interventions—spanning wearable systems, AI applications, VR/AR, smart workstations, advanced ergonomic furniture, and collaborative robotics—collectively contribute to measurable reductions in biomechanical risk, enhanced postural control, and improved worker well‐being [10, 12, 13, 14, 15, 16, 17, 18, 19, 20, 21, 22, 60, 61, 62].

**Figure 2:**
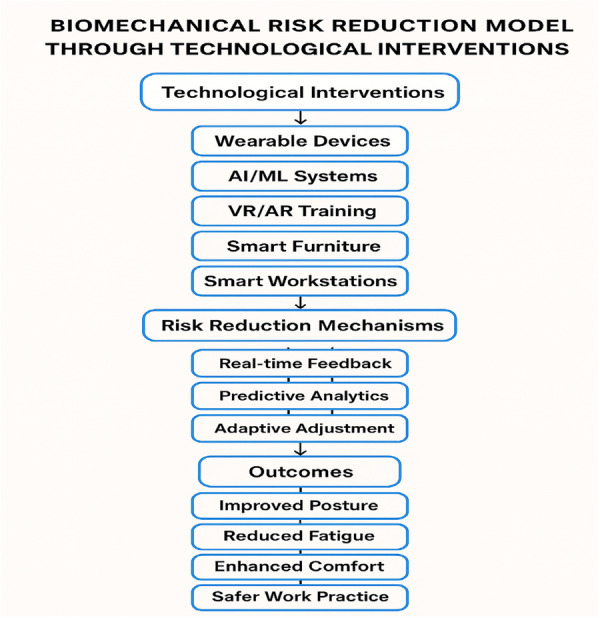
Conceptual model illustrating the pathways of biomechanical risk reduction through six categories of technological ergonomic interventions.

To conceptualize these relationships, a comprehensive Biomechanical Risk Reduction Model was developed (Figure 2), illustrating how each technological domain influences key biomechanical factors (muscular load, postural stress, repetitive motion, cognitive fatigue) through mechanisms such as real‐time feedback, predictive analytics, and adaptive adjustment.

This model visualizes how six technology categories interact with biomechanical risk determinants to yield specific safety and performance outcomes.

Wearables, AI/ML systems, VR/AR tools, smart furniture, intelligent workstations, and collaborative robots activate key reduction mechanisms—enabling proactive mitigation of musculoskeletal risk rather than reactive control [29, 72, 77].

Thus, the framework serves as an analytical foundation for comparing traditional ergonomic approaches with contemporary technological solutions.

### 4.1 Comparison With Traditional Ergonomic Approaches

Conventional ergonomics traditionally relied on expert assessment tools such as RULA and REBA, subjective discomfort surveys, and static workstation design [15, 43]. While these methods effectively identify hazards, they are constrained by observer bias, limited temporal resolution, and the inability to capture dynamic biomechanical data [13, 18].

By contrast, technology‐enhanced ergonomics enables continuous, quantitative, and objective measurement of biomechanical load in real‐world environments [16, 17]. Wearable sensors and AI‐assisted motion tracking replace manual assessment with automated, high‐resolution data, while VR/AR systems support cognitive awareness and posture training [23, 24, 29, 30, 31, 33, 34, 36, 39, 42, 66, 70, 71, 72, 73].

Nevertheless, expert interpretation and participatory ergonomics remain crucial to ensure that digital systems complement rather than replace human insight [25, 28, 69].

The hybrid approach—combining observational expertise with technological feedback—is therefore most effective and consistent with current NIOSH (2022) and OSHA (2023) recommendations for human–technology collaboration [76, 78, 79].

### 4.2 Methodological Quality and Critical Appraisal

The methodological quality of the 74 included studies ranged from moderate to high, yet several weaknesses were identified. Most laboratory‐based experiments offered precise control but limited ecological validity [21, 23, 73], whereas field trials achieved real‐world realism but suffered from lack of standardization and follow‐up [53, 80].

Common issues included small sample sizes, heterogeneity in participant demographics, and limited longitudinal evidence of sustained ergonomic effects [24, 49, 70, 71].

Furthermore, inconsistencies in electromyographic and kinematic measurement calibration across devices introduced bias [44, 74, 76]. Psychosocial dimensions such as perceived workload, stress, and trust in automation were also underexplored [53, 77, 81].

Hence, future research should employ mixed‐method designs integrating biomechanical and psychosocial measures to assess holistic ergonomic impact.

### 4.3 Effectiveness, Limitations, and Potential of Each Technology Within the Model

#### 4.3.1 Wearable Technologies

Effectiveness: Substantial reduction in muscular load—up to 85% in deltoid EMG activity and 12%–15% in spinal extensors—was achieved using passive and active exoskeletons [16, 17].

Limitations: Discomfort, restricted mobility, and heat accumulation limit long‐term adoption [19, 59].

Potential: Integration with AI‐controlled adaptive support and lighter exosuits could optimize biomechanical alignment and reduce user fatigue [29, 64, 72].

#### 4.3.2 AI/ML‐Based Systems

Effectiveness: Automated posture recognition and predictive fatigue detection reduced high‐risk postures by 35% and repetitive strain exposure by 25% [23, 71].

Limitations: Dependence on labeled data, algorithmic bias, and privacy concerns constrain real‐world deployment [28, 70, 75].

Potential: Continuous biomechanical risk mapping and personalized feedback loops could transform proactive ergonomic management [23, 72, 79].

#### 4.3.3 VR/AR Applications

Effectiveness: VR/AR training improved hazard recognition by 40%–41% and enhanced postural stability [30, 31, 32, 33, 34, 35, 39, 42].

Limitations: Visual fatigue, motion discomfort, and limited transferability to real environments remain issues [31, 33, 34].

Potential: Incorporating haptic feedback and live wearable data will bridge the virtual–physical gap for realistic biomechanical learning [36, 41, 82].

#### 4.3.4 Advanced Ergonomic Furniture

Effectiveness: Sit–stand desks and dynamic chairs reduced spinal compression and muscle fatigue by 20%–25% [44, 45, 46].

Limitations: Improper adjustment or poor compliance can nullify benefits [47, 48, 49].

Potential: Pressure‐sensing and automated micro‐adjustment systems can evolve furniture into active biomechanical management tools [50, 52, 53, 75].

#### 4.3.5 Smart Workstations

Effectiveness: Smart workstations decreased time in awkward postures by 29% and improved comfort metrics (*p* < 0.05) [49, 50, 52, 74].

Limitations: High implementation cost and sensor calibration errors challenge scalability [53, 81, 83].

Potential: IoT‐enabled adaptive workstations following NIOSH guidelines can deliver real‐time ergonomic optimization [78, 79, 83].

#### 4.3.6 Human–Robot Collaboration

Effectiveness: Cobots and assistive robots achieved 35%–40% reduction in muscular and spinal load in lifting tasks [54, 56, 59].

Limitations: Cost, safety integration, and dependency risks persist [57, 77, 80, 84].

Potential: Future co‐adaptive robots that dynamically share physical load based on human biomechanical feedback represent a major step in proactive risk reduction [56, 58, 85, 86].

### 4.4 Practical Barriers and Implementation Challenges

Despite strong biomechanical evidence, real‐world deployment faces several barriers:

Economic constraints limit access for SMEs due to high initial and maintenance costs [54, 55, 56, 58, 59]. User acceptance depends on comfort, usability, and participatory design [16, 17, 59, 69]. Data privacy and ethics require clear governance aligned with ILO (2021) and OSHA (2023) frameworks [75, 76, 78, 79]. Training and skill development remain crucial to enable effective human–technology collaboration [53, 83]. Overcoming these barriers demands cross‐sector collaboration between ergonomists, engineers, and organizational leaders.

### 4.5 Human Factors and Ethical Design

Technological ergonomics must prioritize ethical, user‐centered design over mechanical efficiency [63, 77].

Participatory ergonomics—where workers are actively involved in every design phase—ensures trust, comfort, and sustainability [69, 79]. This “human‐in‐the‐loop ergonomics” approach aligns with NIOSH's (2022) call for balancing automation and human judgment [76, 78, 79]. Long‐term success requires assessment of both physical (EMG, posture) and psychosocial (trust, motivation, perceived autonomy) outcomes [81, 84, 85].

### 4.6 Future Directions and Policy Implications

Digital ergonomics is evolving toward precision ergonomics—customizing workplaces to each individual's biomechanical and cognitive profile [65, 87]. To realize this potential, research should:

1. Conduct longitudinal field studies on long‐term biomechanical adaptation [24, 44, 49];
2. Perform cost–benefit analyses to justify technological investment [54, 56, 58];
3. Develop standardized data ethics protocols following ILO/OSHA/NIOSH guidelines [75, 76, 79];
4. Foster interdisciplinary collaboration between ergonomics, robotics, AI, and behavioral sciences [69, 85, 86].

These steps are critical for scaling ethical, sustainable, and effective ergonomic innovation.

### 4.7 Overall Interpretation

Collectively, findings from 74 studies confirm that technology‐enabled ergonomics substantially reduces biomechanical load (12%–85%), improves postural stability (up to 40%), and mitigates fatigue (20%–30%) [10, 12, 13, 14, 15, 16, 17, 18, 19, 20, 21, 22, 43, 46, 47, 51, 60, 61, 62, 74, 84, 88]. However, technology alone cannot eliminate risk; the true value lies in its integration with human expertise and ethical oversight [69, 76, 79]. When implemented under frameworks from ILO, OSHA, and NIOSH, these systems foster a culture of safety, inclusivity, and well‐being, transforming ergonomics from reactive correction to proactive biomechanical risk management [65, 75, 76, 78, 79, 87]. Ultimately, the future of ergonomics is not a replacement of human intelligence with machines but a synergistic partnership between digital innovation and human insight to create healthier, more resilient workplaces [69, 77, 85, 86].

## 5 Conclusion

This narrative review brings together current evidence demonstrating how technology‐driven ergonomics is redefining the prevention of MSDs in contemporary work environments. Drawing on insights from 74 studies, the review shows that emerging technologies—such as wearable sensors, AI, VR/AR, adaptive furniture, smart workstations, and HRC—are collectively transforming ergonomics into a proactive, data‐informed, and human‐centered discipline. The principal contribution of this work lies in its integrative perspective, bridging traditional ergonomic principles with digital innovation through a unified Biomechanical Risk Reduction Framework. Across the reviewed literature, these technologies consistently achieved measurable reductions in muscular load, postural strain, and fatigue, marking significant progress toward safer and more sustainable workplaces.

Nevertheless, several challenges persist. Evidence on long‐term effectiveness, cost‐efficiency, user acceptance, and ethical data governance remains limited. To advance the field, future research should emphasize longitudinal, real‐world studies that evaluate sustained impact, foster interdisciplinary collaboration between engineering, ergonomics, and behavioral sciences, and support the development of standardized frameworks for implementing digital ergonomics. Furthermore, greater attention to psychosocial and cognitive factors—such as trust, engagement, and autonomy—will deepen understanding of how technology can enhance not only biomechanical safety but also holistic well‐being at work.

Ultimately, the future of ergonomics depends on harmonizing human expertise with technological intelligence. Through ethical, inclusive, and adaptive design, tech‐enabled ergonomics can transform workplaces into responsive systems that anticipate risk, promote participation, and nurture long‐term health and resilience in the digital era.

## Author Contributions

Azam Salehi contributed to the conceptualization, methodology, and writing of the manuscript. Azam Salehi participated in data collection, analysis, and manuscript revision. Mahnaz Shakerian was involved in reviewing and editing the manuscript. All authors read and approved the final version of the manuscript.

## Funding

The authors have nothing to report.

## Disclosure

The lead author Azam Salehi affirms that this manuscript is an honest, accurate, and transparent account of the study being reported; that no important aspects of the study have been omitted; and that any discrepancies from the study as planned (and, if relevant, registered) have been explained.

## Ethics Statement

As this paper is a narrative review based on previously published studies, ethical approval was not required.

## Consent

As this paper is a narrative review based on previously published studies, informed consent was not required.

## Conflicts of Interest

The authors declare no conflicts of interest.

## Data Availability

The authors have nothing to report.
